# Virtual Chromoendoscopy Enhancing Visualization of Parasite Anatomical Landmarks: A Case Report

**DOI:** 10.1002/deo2.70438

**Published:** 2026-09-23

**Authors:** Andrés Eduardo Guevara‐Zavala, Sofía Mariño‐Velasco, Jordan Bayne, Joel Omar Jaquez‐Quintana, Héctor J. Maldonado‐Garza, José Alberto González‐González

**Affiliations:** ^1^ Gastroenterology and Endoscopy Service School of Medicine and University Hospital “Dr. José Eleuterio González”, Autonomous University of Nuevo Leon Madero y Gonzalitos Monterrey Mexico

**Keywords:** colonoscopy, helminthiasis, intestinal cestodiasis, in‐vivo diagnosis, virtual chromoendoscopy

## Abstract

Cestodes are parasitic flatworms transmitted through fecal‐oral transmission. Some patients may present with nonspecific gastrointestinal symptoms. Endoscopic identification of intestinal helminths is uncommon and may pose a diagnostic challenge. We describe the endoscopic findings of intestinal cestodiasis and highlight the role of advanced endoscopic imaging techniques in its in vivo recognition. We report the case of a patient with no significant medical history who presented with recent‐onset intermittent gastrointestinal symptoms, including abdominal distension and bloating. Laboratory tests and computed tomography enterography showed no abnormalities. Using magnification and virtual chromoendoscopy, detailed visualization of a parasite attached to the terminal ileal mucosa was achieved. Colonoscopy revealed a thin helminth adherent to the terminal ileal mucosa. Virtual chromoendoscopy enhanced visualization of characteristic anatomical structures. This case highlights the diagnostic value of advanced endoscopic imaging techniques and underscores their usefulness for recognizing uncommon intestinal infections during routine colonoscopy.

## 1 Introduction

Cestodes are parasitic flatworms transmitted through ingestion of undercooked meat, contaminated food or water, or via fecal‐oral transmission [1]. The epidemiology of these zoonotic infections varies depending on geographic region and dietary habits. Most cases are asymptomatic, although some cases may be associated with anemia or malnutrition. Adult cestodes possess a scolex with specialized attachment structures that enable fixation to the intestinal mucosa [2].

## 2 Case Report

We present the case of a 46‐year‐old man with no significant medical history who presented with intermittent postprandial abdominal distension, intermittent diarrhea, diffuse self‐limited abdominal pain, bloating, and borborygmi. He denied any changes in bowel habits, visible gastrointestinal bleeding, weight loss, or constitutional symptoms. Physical examination revealed normal vital signs and a tender abdomen with normal bowel sounds. The three‐sample ova and parasite stool test was negative. Plain abdominal radiographs were unremarkable. Empirical antispasmodic therapy provided no improvement. Contrast‐enhanced computed tomography enterography showed no abnormalities.

**FIGURE 1:**
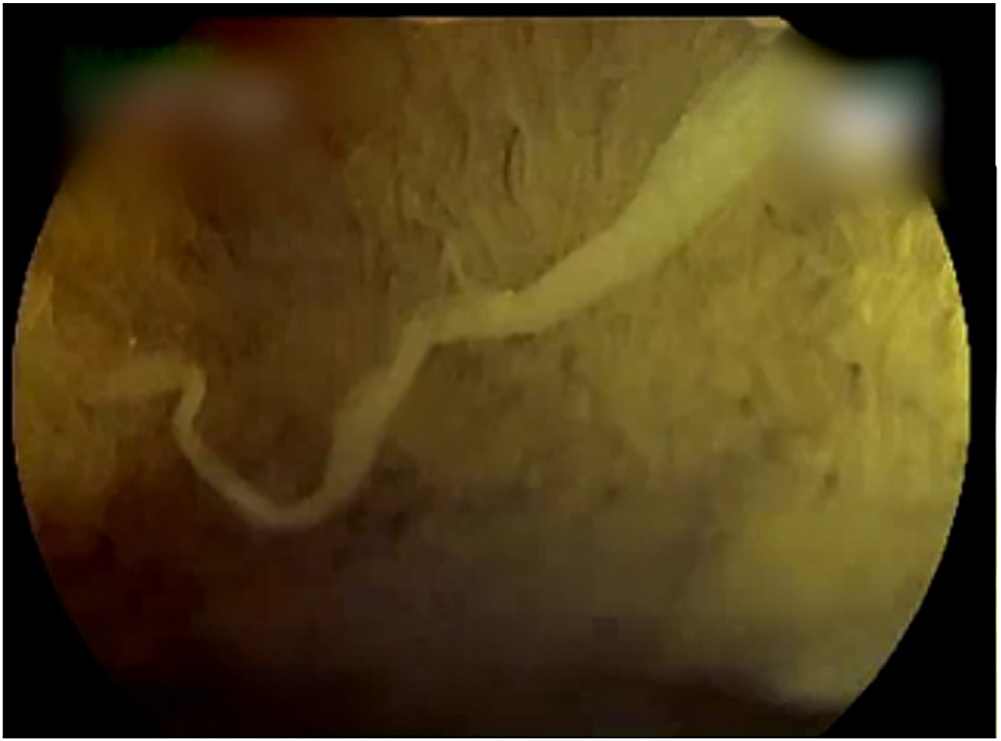
Image obtained under virtual chromoendoscopy showing a mobile, thin, thread‐like structure in the terminal ileum.

**FIGURE 2:**
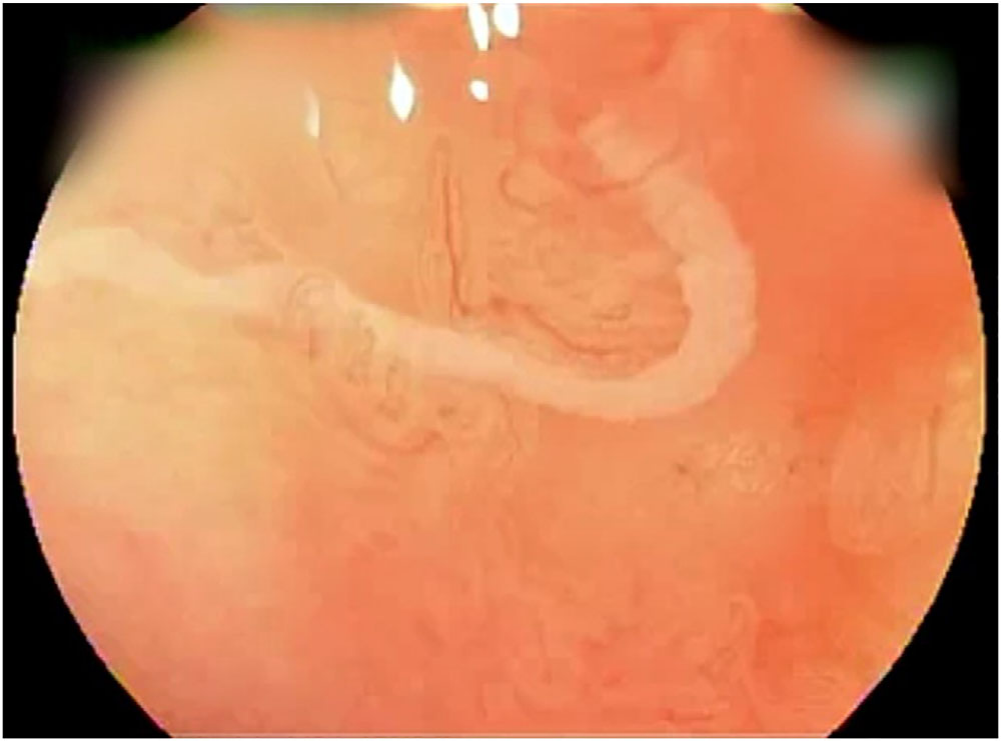
White‐light endoscopic Image showing the cestode.

**FIGURE 3:**
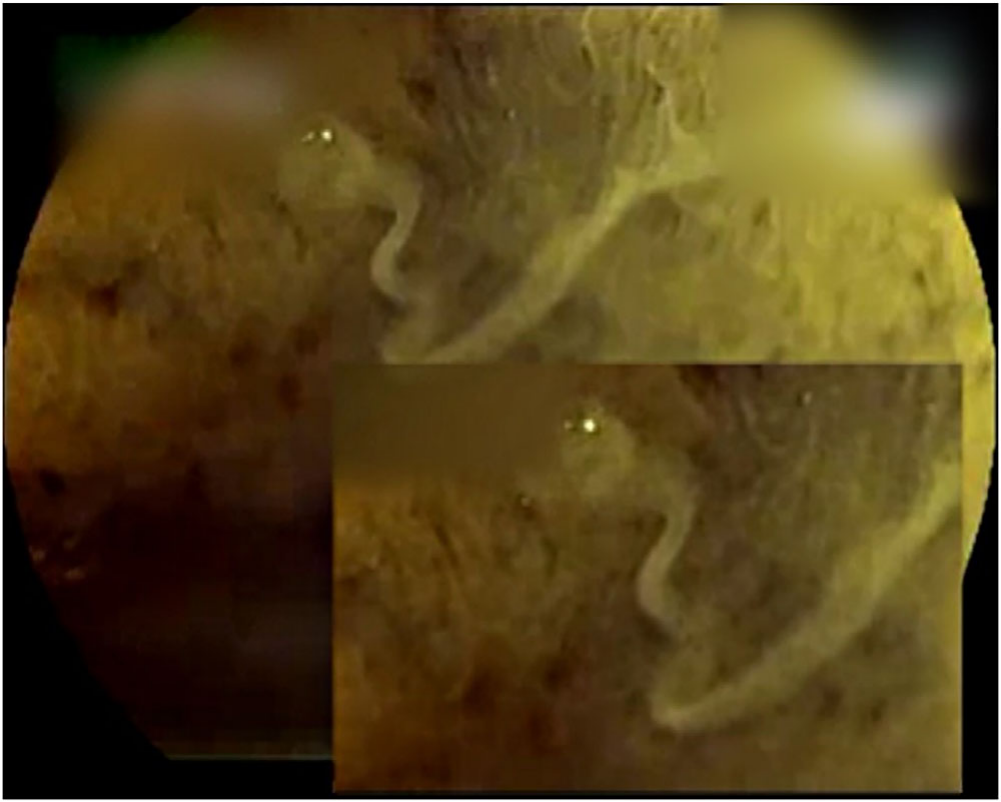
Image obtained under virtual chromoendoscopy: Scolex recognition showing bubble‐like structures corresponding to the suckers.

**FIGURE 4:**
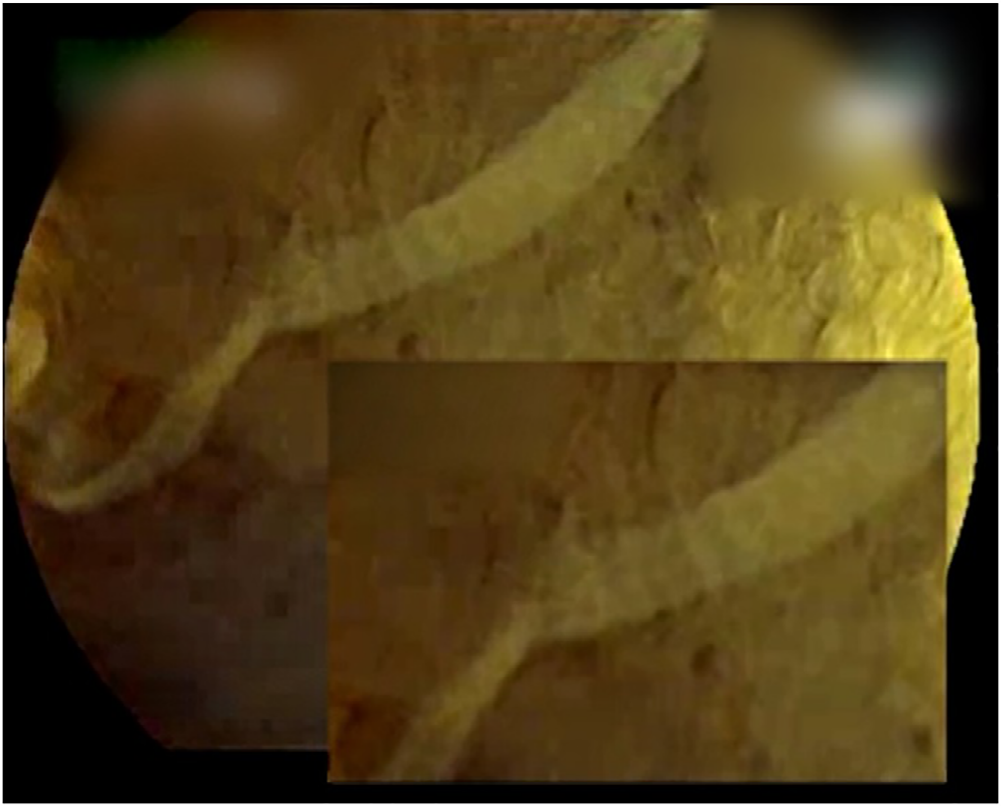
Image obtained under virtual chromoendoscopy: Strobila recognition showing the chain of mature proglottids.

A colonoscopy was subsequently performed; a thin, thread‐like structure was visualized in the terminal ileum (Figures 1, 2). Under magnification, a segmented worm approximately 1 cm in length was identified, adherent to the mucosa. Using virtual chromoendoscopy and magnification FICE (Fuji Intelligent Color Enhancement), the anatomical features of the helminth were clearly enhanced, allowing identification of its three main structures: the scolex (Figure 3), located at the anterior end, including visible suckers used for mucosal attachment, this segment being crucial for species identification; the neck, a non‐segmented, narrow region from which new segments or proglottids differentiate and progressively push older segments distally; and the strobila (Figure 4), the chain of proglottids formed along the body (Video S1). The patient was treated with praziquantel, resulting in symptomatic resolution [3].

## 3 Discussion

The morphological findings were consistent with an intestinal cestode. Two are of principal clinical significance: Taenia (including *T. solium* and *T. saginata*), whose adult forms may reach several meters in length, and *Hymenolepis*, which are considerably smaller. Members of the order *Cyclophyllidea* typically have four suckers on the scolex and may possess a rostellum with hooks; however, certain species lack these structures [4]. In the present case, no rostellar hooks were visualized endoscopically. The absence of visible hooks does not exclude *T. solium*, as visualization may be affected by image resolution, viewing angle, mucus, or parasite motion. Furthermore, the presence of sucker‐like structures alone does not permit species‐level discrimination, as this feature is shared by both *T. solium* and *T. saginata*. Consequently, the endoscopic findings in this case allowed classification at the family level (Taeniidae or Hymenolepididae) but did not support a specific differential diagnosis between genera or species on morphological grounds alone. The parasite was retrieved during the procedure; however, it was not submitted for formal parasitological or molecular analysis, which we recognize as a limitation of the present report. As a result, species‐level identification could not be established, and the endoscopic morphological findings should be interpreted as supportive rather than definitive evidence. This distinction is clinically relevant because *T. solium* is associated with the risk of cysticercosis and neurocysticercosis following autoinfection or environmental contamination [5]. In the present case, no clinical findings suggestive of extraintestinal involvement, including neurological or ocular manifestations, were identified.

The diagnostic pathway in this case highlights a well‐recognized limitation of conventional parasitological workup. Stool examination for ova and parasites, despite its widespread use, carries suboptimal sensitivity for intestinal cestode infections, particularly when proglottid shedding is intermittent or absent at the time of sampling [6]. Similarly, cross‐sectional imaging offers little utility for luminal parasites without associated tissue invasion [7]. The absence of findings on computed tomography enterography and the lack of response to empirical antispasmodic therapy underscore how non‐specific the clinical presentation of intestinal cestode infection can be, frequently leading to diagnostic delay [5]. In this context, ileocolonoscopy with careful inspection of the terminal ileum enabled direct visualization of the parasite and established the diagnosis of intestinal cestode infection. However, definitive species identification could not be achieved because parasitological confirmation was not available [8].

The principal contribution of this case lies in the application of virtual chromoendoscopy with FICE to characterize helminth anatomy in vivo. Standard white‐light endoscopy identified the parasite, but it was the enhanced contrast and optical magnification used by FICE that allowed identification of the scolex, neck, and strobila as distinct structural units [9]. This level of morphological detail has historically required physical extraction of the parasite or histopathological analysis of proglottids. The ability to perform real‐time, non‐invasive morphological characterization directly during the endoscopic procedure represents a meaningful advance, with potential implications for species‐level identification and treatment decision‐making at the point of care [10]. However, FICE‐based morphological characterization does not substitute for parasitological or molecular confirmation when species‐level identification is clinically required and should instead be regarded as a complementary diagnostic adjunct.

Intestinal cestode infections remain underdiagnosed in clinical practice, particularly in regions where dietary and sanitary risk factors are prevalent. This case illustrates that virtual chromoendoscopy is not limited to mucosal pathology assessment; it may serve as a valuable adjunct tool when parasitic infection is encountered or suspected during endoscopic examination.

## Author Contributions

**Andrés Eduardo Guevara‐Zavala**: conceptualization, methodology, writing – review and editing, writing – original draft, and data curation. **Sofía Mariño‐Velasco**: investigation, writing – original draft, and writing – review and editing. **Jordan Bayne**: conceptualization, investigation, and supervision. **Joel Omar Jaquez‐Quintana**: investigation, funding acquisition, and supervision. **Héctor J. Maldonado‐Garza**: investigation, funding acquisition, resources, and supervision. **José Alberto González‐González**: project administration, writing – review and editing, conceptualization, methodology, and validation .

## Funding

The authors have nothing to report.

## Ethics Statement

Ethical committee approval was not required for this single case report according to institutional policies.

## Consent

Written informed consent for publication was obtained from the patient.

## Conflicts of Interest

The authors declare no conflicts of interest.

## Supporting information




**Supporting File**: deo270438‐Supp‐0001‐Video.mp4

## Data Availability

The data that support the findings of this study are available on request from the corresponding author. The data are not publicly available due to privacy or ethical restrictions.
